# Diagnostic accuracy of large-core needle biopsy for nonpalpable breast disease: a meta-analysis

**DOI:** 10.1054/bjoc.1999.1036

**Published:** 2000-02-01

**Authors:** H M Verkooijen, P H M Peeters, E Buskens, V C M Koot, I H M Borel Rinkes, W P ThM Mali

**Affiliations:** Departments of Surgery and Radiology, University Hospital Utrecht, PO Box 85500, Utrecht, GA, 3508, The Netherlands; Julius Center for Patient Oriented Research, Utrecht University Medical School, PO Box 85500, Utrecht, GA, 3508, The Netherlands

**Keywords:** large-core needle biopsy, non-palpable breast disease, diagnostic accuracy, meta-analysis

## Abstract

For the evaluation of non-palpable lesions of the breast, image-guided large-core needle biopsies are increasingly replacing needle-localized open breast biopsies. In this study, the diagnostic accuracy of this minimally invasive technique was evaluated by reviewing the available literature. Five cohort studies were included in a meta-analysis. Sensitivity rate, histological agreement between needle biopsy and subsequent surgery or long-term mammographic follow-up and clinical consequences for different disease prevalences were assessed. The sensitivity rate of large-core needle biopsy for the diagnosis of breast cancer was high (97%). The reclassified agreement rate between core biopsy and subsequent surgical biopsy or long-term mammographic follow-up was also high (94%). In case of 20% breast cancer prevalence among women referred after screening (as in the US), the risk of breast cancer despite benign large-core needle biopsy result is less than 1%. In European countries, however, prevalence of breast cancer among referred women is 60–70%. This would result in a risk of breast cancer despite benign large-core needle biopsy result of 4–6%. The results of this meta-analysis indicate that the image guided large-core needle biopsy is a promising alternative for the needle localized breast biopsy. However, additional research is needed to explore the limiting factors of the technique. Without such detailed knowledge, a benign histological diagnosis on large-core needle biopsy in countries with high prevalence of malignancy among referred women should be interpreted with caution. © 2000 Cancer Research Campaign


British Journal of Cancer (2000) 82(5), 1017–1021
© 2000 Cancer Research Campaign
DOI: 10.1054/ bjoc.1999.1036, available online at http://www.idealibrary.com on IDEAL®

### Diagnostic accuracy of large-core needle biopsy for nonpalpable breast disease: a meta-analysis

**HM Verkooijen[1], PHM Peeters[3], E Buskens[3], VCM Koot[3], IHM Borel Rinkes[1], WPThM Mali[2] and ThJMV van Vroonhoven[1]**

Departments of [1]Surgery and [2]Radiology, University Hospital Utrecht, PO Box 85500, 3508 GA Utrecht, The Netherlands; [3]Julius Center for Patient Oriented Research, Utrecht University Medical School PO Box 85500, 3508 GA Utrecht, The Netherlands

**Summary** For the evaluation of non-palpable lesions of the breast, image-guided large-core needle biopsies are increasingly replacing needle-localized open breast biopsies. In this study, the diagnostic accuracy of this minimally invasive technique was evaluated by reviewing the available literature. Five cohort studies were included in a meta-analysis. Sensitivity rate, histological agreement between needle biopsy and subsequent surgery or long-term mammographic follow-up and clinical consequences for different disease prevalences were assessed. The sensitivity rate of large-core needle biopsy for the diagnosis of breast cancer was high (97%). The reclassified agreement rate between core biopsy and subsequent surgical biopsy or long-term mammographic follow-up was also high (94%). In case of 20% breast cancer prevalence among women referred after screening (as in the US), the risk of breast cancer despite benign large-core needle biopsy result is less than 1%. In European countries, however, prevalence of breast cancer among referred women is 60–70%. This would result in a risk of breast cancer despite benign large-core needle biopsy result of 4–6%. The results of this meta-analysis indicate that the image guided large-core needle biopsy is a promising alternative for the needle localized breast biopsy. However, additional research is needed to explore the limiting factors of the technique. Without such detailed knowledge, a benign histological diagnosis on large-core needle biopsy in countries with high prevalence of malignancy among referred women should be interpreted with caution. © 2000 Cancer Research Campaign

**Keywords:** large-core needle biopsy; non-palpable breast disease; diagnostic accuracy; meta-analysis

One of the consequences of screening for breast cancer and the finding of non-palpable lesions is that it entails needle-localized open breast biopsies. A large proportion of women referred, however, has benign disease. In The Netherlands, the percentage of these benign breast biopsies in the context of non-palpable lesions is 35–40% (Fracheboud et al, 1998). In the USA, 60–90% of the women referred for open breast biopsy are reported to have benign breast disease (Opie et al, 1993; Rubin et al, 1995). Currently, needle-localized open breast biopsy is considered to be the gold standard diagnostic test for non-palpable breast lesions (Burbank and Parker, 1998; Velanovich et al, 1999). Although this procedure is accurate, has a low complication rate and can be performed in a day care setting, most physicians and patients consider it a traumatic one. Moreover, the procedure is expensive; i.e. the costs associated with it represent a major proportion of screening-induced costs (Cyrlak, 1988).

Since the introduction of advanced ultrasound and stereotactic-guided large-core needle biopsy techniques, percutaneous breast biopsies are increasingly replacing open biopsies (Parker, 1994; Fuhrman et al, 1998; Teh et al, 1998). Considering the growing body of literature dealing with these minimally invasive techniques, one might be tempted to conclude that it is already time to replace the needle-localized breast biopsy. But is the scientific evidence adequate to justify adopting the large-core needle biopsy technique?

*Received 24 March 1999*
*Revised 14 September 1999*
*Accepted 18 October 1999*

*Correspondence to:* HM Verkooijen

Because no large randomized controlled trials are available, we set out to review the available literature on percutaneous breast biopsies. A meta-analysis, including well-designed, comparative studies, was performed to assess the diagnostic accuracy of this new procedure.

### MATERIALS AND METHODS

### Reference retrieval and in- and exclusion criteria

A Medline search of the English-language literature published between 1975 and May 1999 was performed. 'Breast', 'biopsy needle', 'diagnosis', 'non(-)palpable' (all subheadings) were used as keywords. A cross-reference search completed the exploration. Because the aim of this study was to assess the diagnostic accuracy of large-core needle biopsy, publications addressing fine-needle aspiration were not eligible for inclusion in the meta-analysis.

Publications were included in the meta-analysis if the pre-set inclusion criteria were met: (1) the mammographic lesions had to be non-palpable; (2) all histological diagnoses of large-core needle biopsy specimens had to be confirmed by either surgical biopsy or adequate follow-up (defined as a minimum of 2 years in at least 90% of the patients); (3) the absolute number of benign and malignant diagnoses had to be derivable; (4) a minimum of five large-core needle biopsy specimens per non-palpable lesion had to be obtained (as has been advocated by Liberman et al (1994)). A total of 118 papers, addressing the issue of large-core needle biopsy for non-palpable breast disease was retrieved. Twenty-five publications were comments, letters or review articles and in 50 studies,

**Table 1** Classification of large-core needle biopsy results as agreements (A), underestimates (U) and overestimates (O) and reclassification for clinical relevance in brackets (adapted from Burbank and Parker, 1998)

| Large-core needle biopsy | Open biopsy/follow-up | | | |
|---|---|---|---|---|
| | Benign disease | ADH | DCIS | Invasive cancer |
| Benign disease | A | U (agreement) | U (miss) | U (miss) |
| ADH | O (agreement) | A | U (ADH underestimate) | U (ADH underestimate) |
| DCIS | O (agreement) | O (agreement) | A | U (DCIS underestimate) |
| Invasive cancer | O (agreement) | O (agreement) | O (agreement) | A |

ADH, atypical ductal hyperplasia; DCIS, ductal carcinoma in situ.

the diagnostic performance of large-core needle biopsy was not the object of study. Of the 43 publications addressing the diagnostic accuracy of large-core needle biopsy, five publications were included in the meta-analysis (Parker et al, 1991; Elvecrog et al, 1993; Gisvold et al, 1994; Pijnappel et al, 1997; Jackman et al, 1999). (A list of the 38 excluded publications and the reasons for exclusion is available upon request.) Thirty studies were excluded because the histological diagnoses on needle biopsy were not satisfactorily confirmed. Six studies were excluded because an average of fewer than five core biopsies per lesion was obtained. In one paper, the absolute number of non-palpable lesions was not derivable and one paper was excluded because the absolute number of benign and malignant lesions was not derivable. Two of the authors (HMV and VCMK) independently extracted the data from the studies using a standard form. In case of discrepancies consensus was reached.

### Meta-analysis of diagnostic accuracy

The diagnostic accuracy of large-core needle biopsy was assessed using a method introduced by Burbank and Parker (1998). For this purpose, the results of the studies were collapsed into a four by four table. Firstly, the histological outcomes from the needle biopsy procedures were classified according to one of the following four categories:

1. benign breast disease (including normal breast tissue)
2. atypical ductal hyperplasia (ADH) (This category also includes other high-risk lesions, e.g. lobular carcinoma in situ, atypical lobular hyperplasia and radial scar. Because ADH is the most common of these lesions, this category was named accordingly
3. ductal carcinoma in situ (DCIS)
4. infiltrating breast cancer.

Lesions that were surgically removed were divided into the same four categories according to the histological diagnosis. Lesions with a benign histological large-core needle biopsy result that were not surgically removed and that remained unchanged during follow-up, were categorized as benign.

Then, the cells in the four by four table were initially labelled as histological agreement cells, underestimate or overestimate cells (Table 1). Agreement cells were defined as cells with identical pathology at large-core needle biopsy and open biopsy or follow-up. Cells, indicating a higher degree of pathology at large-core needle biopsy than at open biopsy, were classified as overestimates. Underestimate cells indicate a lower degree of pathology at large-core needle biopsy than at open biopsy.

The underestimate cells were divided into three subcategories. The DCIS underestimate rate was defined as the percentage of DCIS lesions on large-core needle biopsy that is upgraded to invasive cancer in the surgical specimen.

The ADH underestimate rate was defined as the percentage of ADH lesions on large-core needle biopsy that is upgraded to DCIS or invasive cancer. The reason that these outcomes were classified as underestimates rather than misses is that a diagnosis of ADH on large-core needle biopsy is always an indication for surgical biopsy. Atypical ductal hyperplasia is a benign disease known to be associated with an increased risk of breast cancer (Marshall et al, 1997; Page et al, 1998; Tavassoli, 1998). Several studies have already demonstrated that when ADH is diagnosed on large-core needle biopsy, the risk of malignancy at surgical biopsy is 33–52% (Liberman et al, 1995; Gadzala et al, 1997; Moore et al, 1997; Fuhrman et al, 1998). Similarly, histological diagnoses of radial scar, lobular carcinoma in situ and atypical lobular hyperplasia on large-core needle biopsy are often associated with invasive or in situ carcinomas (Lee et al, 1997; Liberman et al, 1997; Brown et al, 1998; Fuhrman et al, 1998), and therefore always an indication for surgical biopsy.

The miss rate was defined as the proportion of all breast cancers (invasive cancer and DCIS) with a diagnosis of only benign disease on large-core needle biopsy. Accordingly, the sensitivity rate was defined as one minus the miss rate.

The remaining overestimate and underestimate cells were then reclassified into clinically relevant categories (Table 1). For clinically relevant purposes, a diagnosis of benign disease on large-core needle biopsy, upgraded to ADH, was reclassified as agreement. Although the finding of ADH in a surgical specimen is associated with an increased risk of breast cancer (Marshall et al, 1997; Page et al, 1998; Tavassoli, 1998), it does not have clinical consequences. Moreover, ADH lesions are by definition small (≤2 mm) (Tavassoli, 1998) and therefore the finding of ADH in a surgical specimen is nearly always incidental. In addition, Burbank and Parker (1998) argue that overestimates are actually clinically relevant agreements rather than disagreements. For example, if large-core needle biopsy had identified invasive breast cancer and the surgical specimen contained only fibrocystic changes, the target lesion would still maintain the diagnosis of invasive cancer. The lower degree of pathology seen in the open

© 2000 Cancer Research Campaign

**Table 2** Characteristics of the studies included in meta-analysis

| First author year of publication | Imaging technique | Needle diameter | Consecutive patients | Mean age | Number of biopsy procedures (*n*) | Proportion microcalci-fications | Proportion DCIS and invasive cancer | Proportion DCIS of all malignancies | Complications |
|---|---|---|---|---|---|---|---|---|---|
| Parker 1991 | Stereotaxis | 14-gauge | ? | ? | 102 | ? | 23% | 9% | 0 |
| Elvecrog 1993 | Stereotaxis | 14-gauge | Cons[a] | ? | 100 | 26% | 35% | 11% | 1 |
| Gisvold 1994 | Stereotaxis | 14-gauge | Non-cons[a] | 59 | 104 | 33% | 43% | ? | 1 |
| Pijnappel[b] 1997 | Stereotaxis and ultrasound | 14-gauge | Cons[a] | 55 | 76 | 21% | 57% | 30% | 0 |
| Jackman 1999 | Stereotaxis | 14-gauge | Cons[a] | 55 (median) | 483 | 48% | 31% | 40% | ? |

[a]Cons = consecutive patients. [b]All studies were conducted in the USA, except for the study of Pijnappel et al, which was conducted in The Netherlands.

**Table 3** Number of DCIS and ADH and underestimate rates (95% CI)

| Study | DCIS on needle biopsy | DCIS-lesions upgraded to invasive cancer | DCIS underestimate rate (%) | ADH on needle biopsy | ADH lesions upgraded to carcinoma | ADH underestimate rate (%) |
|---|---|---|---|---|---|---|
| Parker 1991 | 2 | 0 | 0 (0–84) | ? | ? | ? |
| Elvecrog 1993 | ? | ? | ? | 6 | 0 | 0 (0–52) |
| Gisvold 1994 | ? | ? | ? | 2 | 1 | 50 (13–99) |
| Pijnappel 1997 | 13 | 3 | 23 (5.0–54) | 7 | 1 | 14 (4.0–58) |
| Jackman 1999 | 56 | 8 | 14 (6.0–26) | 30 | 16 | 53 (34–72) |
| Pooled | 71 | 11 | 15 (8.0–26) | 45 | 18 | 40 (26–56) |

DCIS, ductal carcinoma in situ; ADH, atypical ductal hyperplasia.

biopsy specimen can be explained by either complete removal of the lesion by the large-core needle biopsy (Dronkers, 1992; Mikhail et al, 1994) or by inadequate surgical excision (lesion not removed). Therefore, these overestimate cells were also reclassified as agreement cells. Accordingly, the reclassified agreement rate was defined as the proportion of cells not classified as DCIS underestimate, ADH underestimate or miss.

Underestimate rates, miss rates and reclassified agreement rates were calculated as described above for each of the five studies as well as pooled estimates after testing for homogeneity of the studies using Fisher exact test (Altman, 1991).

Finally, the clinical consequences of the miss rate of the large-core needle biopsy technique were evaluated for different disease prevalences. For this purpose, the predictive value of a benign biopsy result was calculated for different disease prevalences, applying the following formula:

Risk of malignancy despite benign histological diagnosis on large-core needle biopsy =
(1−sensitivity)*prevalence/((1−sensitivity)*prevalence + specificity*(1−prevalence))

As discussed previously, a malignant diagnosis on large-core needle biopsy followed by a benign diagnosis of the open biopsy specimen was not considered as an overestimate or false-positive result. We therefore agreed on a specificity rate of the large-core needle biopsy of 1.0. Statistics were performed using Statistical Package for Social Sciences 6.0 (SPSS Inc. Chicago, IL, USA). Exact confidence intervals were calculated.

### RESULTS

Table 2 shows the data obtained from the five papers. In all studies, stereotactic guidance was applied. Pijnappel et al (1997) used ultrasound guidance in addition to stereotactic guidance in three cases (4%). Stereotactic biopsy was performed with the patient in prone position by using a 14-gauge needle and a long throw biopsy gun. In four studies all histological diagnoses of large-core needle biopsy specimens were confirmed by additional open biopsy and generally these two procedures took place on the same day (Parker et al, 1991; Elvecrog et al, 1993; Gisvold et al, 1994; Pijnappel et al, 1997). In the study of Jackman et al (1999), 295 patients (61%) with a benign diagnosis on large-core needle biopsy did not undergo immediate open biopsy, but mammographic follow-up. During follow-up, repeat biopsy was performed in 36 patients. Of the remaining 259 patients, 96% was followed for at least 2 years and the median follow-up period was 55 months. Lesions with discrepancy between mammography and histological diagnosis on large-core needle biopsy were surgically removed. Also, patients with a diagnosis of atypical ductal or lobular hyperplasia, radial scar or carcinoma (invasive and in situ) on large-core needle biopsy were planned for surgery.

A total of 865 large-core needle biopsy procedures was performed and 56% was carried out in the context of one study (Jackman et al, 1999). In four studies the percentage of lesions with microcalcifications was reported (Elvecrog et al, 1993; Gisvold et al, 1994; Pijnappel et al, 1997; Jackman et al, 1999) which varied from 21 to 48%. The malignancy rate varied from 23 to 57% and four studies reported that 9–40% of all malignant

*© 2000 Cancer Research Campaign*

**Table 4** Total number of malignancies (i.e. invasive and DCIS) and sensitivity (95% CI)

| Study | Total number of malignancies | Number of malignancies missed at large-core needle biopsy | Sensitivity % |
|---|---|---|---|
| Parker 1991 | 23 | 1 | 96 (78–100) |
| Elvecrog 1993 | 35 | 1 | 97 (85–100) |
| Gisvold 1994 | 45 | 3 | 93 (82–99) |
| Pijnappel 1997 | 43 | 1 | 98 (88–100) |
| Jackman 1999 | 161 | 2 | 99 (96–100) |
| Pooled | 307 | 8 | 97 (95–99) |

**Table 5** Risk of malignancy despite benign histological diagnosis on large-core needle biopsy as a function of prevalence of malignancy (sensitivity rate 97%, specificity rate 100%)

| Prevalence of malignancy (%) | Risk of malignancy despite benign needle biopsy result (%) |
|---|---|
| 10 | 0.3 |
| 20 | 0.6 |
| 30 | 1.3 |
| 40 | 2.0 |
| 50 | 2.9 |
| 60 | 4.3 |
| 70 | 6.5 |

lesions was DCIS only. The number of complications was low; only one haematoma and one infection (which also might have been caused by the open biopsy procedure) were described.

Table 3 presents the DCIS and ADH underestimate rates. The DCIS underestimate rate was derivable for three of five studies and the pooled DCIS underestimate rate of these three studies was 15% (95% CI 8–26%). The ADH underestimate rate could be derived for four studies and the pooled ADH underestimate rate was 40% (95% CI 26–56%).

In Table 4 the sensitivity rate of large-core needle biopsy for all studies was calculated. Of 307 carcinomas, eight were diagnosed as benign by large-core needle biopsy (3%). Consequently, the sensitivity rate was 97% (95% CI 95–99%).

The reclassified agreement rate was derivable for two studies. In the study of Pijnappel et al (1997), there was clinically relevant discrepancy in five of 76 cases (one ADH underestimate, three DCIS underestimates and one miss), resulting in a reclassified agreement rate of 93% (95% CI 85–98%). In the study of Jackman et al (1999) there were 26 discrepancies (16 ADH underestimates, eight DCIS underestimates and two misses) on a total of 483 lesions, resulting in a reclassified agreement rate of 95% (95% CI 92–97) in this study. Combining the results of these two studies gives rise to a pooled reclassified agreement rate of 94% (95% CI 92–96%).

Table 5 shows the risk of malignancy despite benign large-core needle biopsy result for different assumptions of breast cancer prevalences. In a population with a low prevalence of malignancy of e.g. 20% (i.e. USA), the probability of a carcinoma being present despite benign needle biopsy result is low (0.6%). In Western Europe, however, 60–70% of the lesions detected by screening and referred for histological biopsy turn out to be malignant, due to the use of different cut-off points for referral. If in such a setting a large-core needle biopsy reveals benign disease, the probability of a carcinoma being present will still be 4–6%.

### DISCUSSION

This meta-analysis showed that the diagnostic accuracy of large-core needle biopsy is high. The sensitivity rate was 97% and the reclassified agreement rate was 94%. Accordingly, large-core needle biopsy seems to be an attractive alternative for the needle-localized open breast biopsy.

We used the approach of Burbank and Parker (1998) for evaluating the diagnostic performance of large-core needle biopsy. With this approach, the reclassified agreement rate is not an estimate for exact histological accordance between large-core needle biopsy and surgical biopsy. Certain discrepancies between large-core needle biopsy and open biopsy (e.g. fibrocystic disease on large-core needle biopsy and ADH on surgical biopsy, or DCIS on large-core needle biopsy and fibrocystic disease on open biopsy) were considered as agreements. The reclassified agreement rate is therefore a clinically relevant and pragmatic estimate for the accordance between large-core needle biopsy and actual disease status. This estimate, however, could only be calculated for two studies.

A DCIS underestimate rate of 15% indicates that 15% of the patients with a diagnosis of DCIS on large-core needle biopsy will prove to have invasive breast cancer at surgery. In most of these cases, an additional surgical procedure will then be necessary (axillary dissection with or without re-excision). Similarly, all patients with a diagnosis of ADH, atypical lobular hyperplasia, lobular carcinoma in situ or radial scar on large-core needle biopsy need to undergo open breast biopsy. Although the use of large-core needle biopsy eventually results in a correct histological diagnosis in these patient categories, large-core needle biopsy is an extra diagnostic procedure as compared to the situation with open breast biopsy as initial diagnostic procedure.

The pooled analysis showed a sensitivity rate of 97%. This value may be overestimated due to the non-blindness of the pathologists (Irwig et al, 1994). In four studies large-core needle biopsy procedures as well as open biopsy procedures were performed on the same day (Parker et al, 1991; Elvecrog et al, 1993; Gisvold et al, 1994; Pijnappel et al, 1997). Therefore, pathologists may not have been blinded for the results of the reference standard test (open biopsy).

Moreover, 56% of all lesions were derived from the study of Jackman et al (1999). The relatively high sensitivity rate reported in this study has therefore substantially influenced the results of this meta-analysis.

Cut-off points for referral after breast cancer screening differ substantially between the USA and Europe. As a consequence, prevalence of malignancy among women referred for breast biopsy is approximately 20% in the USA compared to 60% in Europe. This difference can be explained by the fact that screening for breast cancer is more difficult in the USA than it is in Europe. In the first place, every American woman over 40 years is advised to undergo annual screening mammography, while in The Netherlands breast cancer screening starts at the age of 50. Because breast tissue is denser in younger women, screening mammograms will be more difficult to interpret, resulting in a lower sensitivity and specificity rate (Beemsterboer et al, 1998; UK Trial of Early Detection of Breast Cancer Group, 1999). Secondly, in the USA, approximately 50% of post-menopausal women use hormone replacement therapy (HRT) (Grodstein et al, 1997), compared to only 22% in the UK (Achuthan et al, 1999).

 © 2000 Cancer Research Campaign

HRT influences the breast parenchyma, resulting in a decreased sensitivity and specificity rate of screening mammographies (Laya et al, 1996).

The difference in prevalence of malignancy leaves the practical consequences of replacing open biopsy by large-core needle biopsy open to discussion. In the USA, the need for minimally invasive and less expensive diagnostic tests is high because a large proportion of women undergoes benign breast biopsy. The risk of breast cancer in case of benign large-core core needle biopsy results is small (0.6%) in a population with relatively low breast cancer prevalence. On the contrary, in European screening settings the higher breast cancer prevalence among referred women will result in a relatively high risk of breast cancer in case of benign large-core needle biopsy (4–6%). One might argue which procedure is more effective in diagnosing breast cancer and preventing mortality of it: a rather unselective referral of women with low disease prevalence, worked up by the large-core needle procedure, or a more restricted selection of women with a relatively high disease prevalence, worked up by needle-localized open breast biopsy.

In addition, the miss rate of 3% may be reduced by identifying other high-risk categories, besides ADH, atypical lobular hyperplasia, lobular carcinoma in situ and radial scars. Perhaps the category of microcalcification lesions should be handled more cautiously, as it has been suggested that the miss rate of stereotactic large-core needle biopsy is higher for microcalcifications than for mass lesions (Burbank and Parker, 1998).

In The Netherlands a prospective multicentre study (funded by the Ministry of Public Health) was started at the end of 1997 to address these issues. The study aims to include 1000 consecutive patients with non-palpable breast lesions who will all undergo both stereotactically guided large-core needle biopsy (14-gauge with a long-throw biopsy gun) on a prone table and surgical breast biopsy. Preferences of women and cost consequences will also be taken into account.

### CONCLUSION

The high sensitivity rate (97%) and high reclassified agreement rate (94%) of the large-core needle biopsy make this technique a promising alternative for needle-localized open breast biopsy. However, additional research is required to elucidate limiting factors of the technique and to decide on optimal patient selection strategies. Without such detailed knowledge, a benign histological diagnosis on large-core needle biopsy in countries with high prevalence of malignancy among referred women should be handled with caution.

### REFERENCES

Achuthan R, Parkin G and Horgan K (1999) Screening mammography for women starting hormone replacement therapy. *Lancet* **353**: 1855

Altman DG (1991) Comparing groups–categorical data. In: *Practical Statistics for Medical Research*, Altman DG, pp. 229–272. Chapman and Hall: London

Beemsterboer PMM, Warmerdam PG, Boer R and Koning de HJ (1998) Radiation risk of mammography related to benefit in screening programmes: a favourable balance? *J Med Screen* **5**: 81–87

Brown TA, Wall JW, Christensen ED, Smith DV, Holt CA, Carter PL, Patience TH, Babu SS and Williard W (1998) Atypical hyperplasia in the era of stereotactic core needle biopsy. *J Surg Oncol*. **67**: 168–173

Burbank F and Parker SH (1998) Methods for evauating the quality of an image-guided breast biopsy program. *Semin Breast Dis* **1**: 71–83

Cyrlak D (1988) Induced costs of low-cost screening mammography. *Radiology* **168**: 661–663

Dronkers DJ (1992) Stereotaxic core biopsy of breast lesions. *Radiology* **183**: 631–634

Elvecrog EL, Lechner MC and Nelson MT (1993) Nonpalpable breast lesions: correlation of stereotaxic large-core needle biopsy and surgical biopsy results. *Radiology* **188**: 453–455

Fracheboud J, Koning de HJ, Beemsterboer PMM, Boer R, Hendriks JHCL, Verbeek ALM, Ineveld v BM, Bruyn de AE and Maas van der PJ (1998) Nationwide breast cancer screening in The Netherlands: results of initial and subsequent screening 1990–1995. *Int J Cancer* **75**: 694–698

Fuhrman GM, Cederbom GJ, Bolton JS, King TA, Duncan JL, Champaign JL, Smetherman DH, Farr GH, Kuske RR and McKinnon WM (1998) Image-guided core-needle breast biopsy is an accurate technique to evaluate patients with nonpalpable imaging abnormalities. *Ann Surg* **227**: 932–939

Gadzala DE, Cederbom GJ, Bolton JS, McKinnon WM, Farr GH, Champaign J, Ordoyne K, Chung K and Fuhrman GM (1997) Appropriate management of atypical ductal hyperplasia diagnosed by stereotactic core needle breast biopsy. *Ann Surg Oncol* **4**: 283–286

Gisvold JJ, Goellner JR, Grant CS, Donohue JH, Sykes MW, Karsell PR, Coffey SL and Sin-ho J (1994) Breast biopsy: a comparative study of stereotaxically guided core and excisional techniques. *Am J Radiol* **162**: 815–820

Grodstein F, Stampfer MJ and Colditz GA (1997) Postmenopausal hormone therapy and mortality. *N Engl J Med* **336**: 1769–1775

Irwig L, Tosteson ANA, Gatsonis C, Lau J, Colditz G, Chalmers TC and Mosteller F (1994) Guidelines for meta-analyses evaluating diagnostic tests. *Ann Intern Med* **120**: 667–676

Jackman RJ, Nowels KW, Rodriguez-Soto J, Marzoni FA, Finkelstein SD and Shephard MJ (1999) Stereotactic, automated, large-core needle biopsy of nonpalpable breast lesions: false-negative and histologic underestimate rates after long-term follow up. *Radiology* **210**: 799–805

Laya MB, Larson EB, Taplin SH and White E (1996) Effect of estrogen replacement therapy on the specificity and sensitivity of screening mammography. *J Natl Cancer Inst* **88**: 643–649

Lee CH, Egglin TK, Philpotts LE, Mainiero MB and Tocino I (1997) Cost-effectiveness of stereotactic core needle biopsy: analysis by means of mammographic finding. *Radiology* **202**: 849–854

Liberman L, Cohen MA, Dershaw DD, Abramson AF, Hann LE and Rosen PP (1995). Atypical ductal hyperplasia diagnosed at stereotaxic core biopsy of breast lesions; an indication for surgical biopsy. *Am J Radiol* **164**: 1111–1113

Liberman L, Dershaw DD, Glassman JR, Abramson AF, Morris EA, LaTrenta LR and Rosen PP (1997) Analysis of cancers not diagnosed at stereotactic core breast biopsy. *Radiology* **203**: 151–157

Marshall LM, Hunter DJ, Connolly JL, Schnitt SJ, Byrne CB, London SJ and Colditz GA (1997) Risk of breast cancer associated with atypical hyperplasia of lobular and ductal types. *Cancer Epidemiol Biomarkers Prev* **6**: 297–301

Mikhail RA, Nathan RC, Weiss M, Tummala RM, Mullangi UR, Lawrence L and Mukkamala A (1994) Stereotactic core needle biopsy of mammographic breast lesions as a viable alternative to surgical biopsy. *Ann Surg Oncol* 1: 363–367

Moore MM, Hargett CW, Hanks JB, Fajardo LL, Harvey JA, Frierson HF and Slingluff CL (1997) Association of breast cancer with the finding of atypical ductal hyperplasia at core breast biopsy. *Ann Surg* **225**: 726–733

Opie H, Estes NC, Jewell WR, Chang CHJ, Thomas JA and Estes MA (1993) Breast biopsy for nonpalpable lesions: a worthwhile endeavor? *Am Surg* **59**: 490–494

Page DL, Jensen RA and Simpson JF (1998) Premalignant and malignant disease of the breast: the roles of the pathologist. *Mod Pathol* **11**: 120–128

Parker SH (1994) Percutaneous large core breast biopsy. *Cancer* **74**: 256–262

Parker SH, Lovin JD, Jobe WE, Burke BJ, Hopper KD and Yakes WF (1991) Nonpalpable breast lesions: stereotactic automated large-core biopsies. *Radiology* **180**: 403–407

Pijnappel RM, Dalen van A, Borel Rinkes IHM, Tweel van den JG and Mali WPThM (1997) The diagnostic accuracy of core biopsy in palpable and non-palpable breast lesions. *Eur J Radiol* **24**: 120–123

Rubin E, Dempsey PJ, Pile NS, Bernreuter WK, Urist MM, Shumate CR and Maddox WA (1995) Needle-localization biopsy of the breast: impact of a selective core needle biopsy program on yield. *Radiology* **195**: 627–631

Tavassoli FA (1998) Ductal carcinoma in situ: introduction of the concept of ductal intraepithelial neoplasia. *Mod Pathol* **11**: 140–154

Teh WL, Evans AJ and Wilson ARM (1998) Definitive non-surgical breast diagnosis: the role of the radiologist. *Clin Radiol* **53**: 81–84

UK Trial of Early Detection of Breast Cancer Group (1999) 16-year mortality from breast cancer in the UK Trial of Early Detection of Breast Cancer. *Lancet* **353**: 1909–1914

Velanovich V, Lewis FR, Nathanson SD, Strand VF, Talpos GB, Bhandarkar S, Elkus R, Szymanski W and Ferrara JJ (1999) Comparison of mammographically guided breast biopsy techniques. *Ann Surg* **229**: 625–633

© 2000 Cancer Research Campaign
